# Cytochrome b5 and Cytokeratin 17 Are Biomarkers in Bronchoalveolar Fluid Signifying Onset of Acute Lung Injury

**DOI:** 10.1371/journal.pone.0040184

**Published:** 2012-07-06

**Authors:** Antoine Ménoret, Sanjeev Kumar, Anthony T. Vella

**Affiliations:** University of Connecticut Health Center, Farmington, Connecticut, United States of America; Montana State University, United States of America

## Abstract

Acute lung injury (ALI) is characterized by pulmonary edema and acute inflammation leading to pulmonary dysfunction and potentially death. Early medical intervention may ameliorate the severity of ALI, but unfortunately, there are no reliable biomarkers for early diagnosis. We screened for biomarkers in a mouse model of ALI. In this model, inhalation of *S. aureus* enterotoxin A causes increased capillary permeability, cell damage, and increase protein and cytokine concentration in the lungs. We set out to find predictive biomarkers of ALI in bronchoalveolar lavage (BAL) fluid before the onset of clinical manifestations. A cutting edge proteomic approach was used to compare BAL fluid harvested 16 h post *S. aureus* enterotoxin A inhalation *versus* BAL fluid from vehicle alone treated mice. The proteomic PF 2D platform permitted comparative analysis of proteomic maps and mass spectrometry identified cytochrome b5 and cytokeratin 17 in BAL fluid of mice challenged with *S. aureus* enterotoxin A. Validation of cytochrome b5 showed tropic expression in epithelial cells of the bronchioles. Importantly, *S. aureus* enterotoxin A inhalation significantly decreased cytochrome b5 during the onset of lung injury. Validation of cytokeratin 17 showed ubiquitous expression in lung tissue and increased presence in BAL fluid after *S. aureus* enterotoxin A inhalation. Therefore, these new biomarkers may be predictive of ALI onset in patients and could provide insight regarding the basis of lung injury and inflammation.

## Introduction

Pulmonary biomarkers are needed to predict the clinical course of lung disease, status, progression, and response to treatment [1], [2]. A key aspect in biomarker discovery is uncovering molecules that appear early during disease initiation, when the natural history of the disease can be modified. During acute lung injury (ALI), several factors correlate with tissue damage such as recruitment of neutrophils and macrophages, and also increases in IFNγ in BAL fluid [3]. However, these events occur late in the disease process, once ALI is fully initiated. Therefore, it is critical to find new biomarkers that occur prior to tissue damage [4] and perhaps even before cytokines are produced.

In humans, a major cause of ALI is the immune response to a lung infection [5], [6]. Naturally there are other causes such as exposure to aerosolized toxic chemicals, aspiration, and multiple trauma. However, even in these cases, a secondary infection may trigger the inflammatory response that leads to ALI [6]. Although no animal model is a perfect representation of ALI in humans [7], bacterial lipopolysaccharide (LPS) does induce key features such as alveolitis, neutrophil recruitment, and induction of IFNγ that mimic symptoms of human ALI. However, unlike infection with whole organisms, LPS does not specifically stimulate T cells, which likely make a substantial contribution to disease in humans. Therefore, pathogen byproducts that stimulate T cells add a critical dimension to the modeling of human ALI.

*Staphylococcus aureus* secretes enterotoxin proteins that are pathogenic in humans, can cause toxic shock syndrome [4], and are implicated in Chronic Obstructive Pulmonary Disease (COPD) [8]. Importantly, *S. aureus* has been detected in nasal polyps of patients suffering from chronic rhinosinusitis, and IgE antibodies specific to *S. aureus* enterotoxin A (SEA) have been detected in patients with severe asthma, suggesting that SEA is involved in the pulmonary immune response [9], [10]. In our previous mouse study we showed that inhalation of SEA induced T cells to migrate into lung, become effectors, and prime innate cells [11]. This response was rapid, marked by neutrophil recruitment and increases of protein in BAL fluid along with high levels of IFNγ and alveolitis. Consequently, the SEA inhalation model approximates many aspects of human ALI.

Our goal was to use a proteomic mining strategy to uncover differences in BAL fluid from SEA treated mice *versus* vehicle alone controls. The PF 2D proteomic platform allows two-dimensional liquid fractionation of biological fluid based on isoelectric focusing and hydrophobicity. We used this strategy to detect changes during colon cancer chemoprevention [12], and others have used it for biomarker discovery [13], [14]. Recently, this method uncovered a pathway involved in cytokine based inflammation [15]. Therefore, we set out to find distinct changes in the BAL fluid proteome of SEA treated mice compared to vehicle alone, and found an unexpected increase in microsomal cytochrome b5 and cytokeratin 17. These data were validated and coincided with rapid changes in lung pathology that included increased lung inflammation. Thus, our mouse model data show that cytochrome b5 and cytokeratin 17 are detected in BAL fluid very early after lung damage, and thus may be potential biomarkers of pulmonary injury in patients with ALI or other life-threatening diseases.

## Results

### Inhalation of SEA Induces Lung Inflammation and Damage

**Figure 1:**
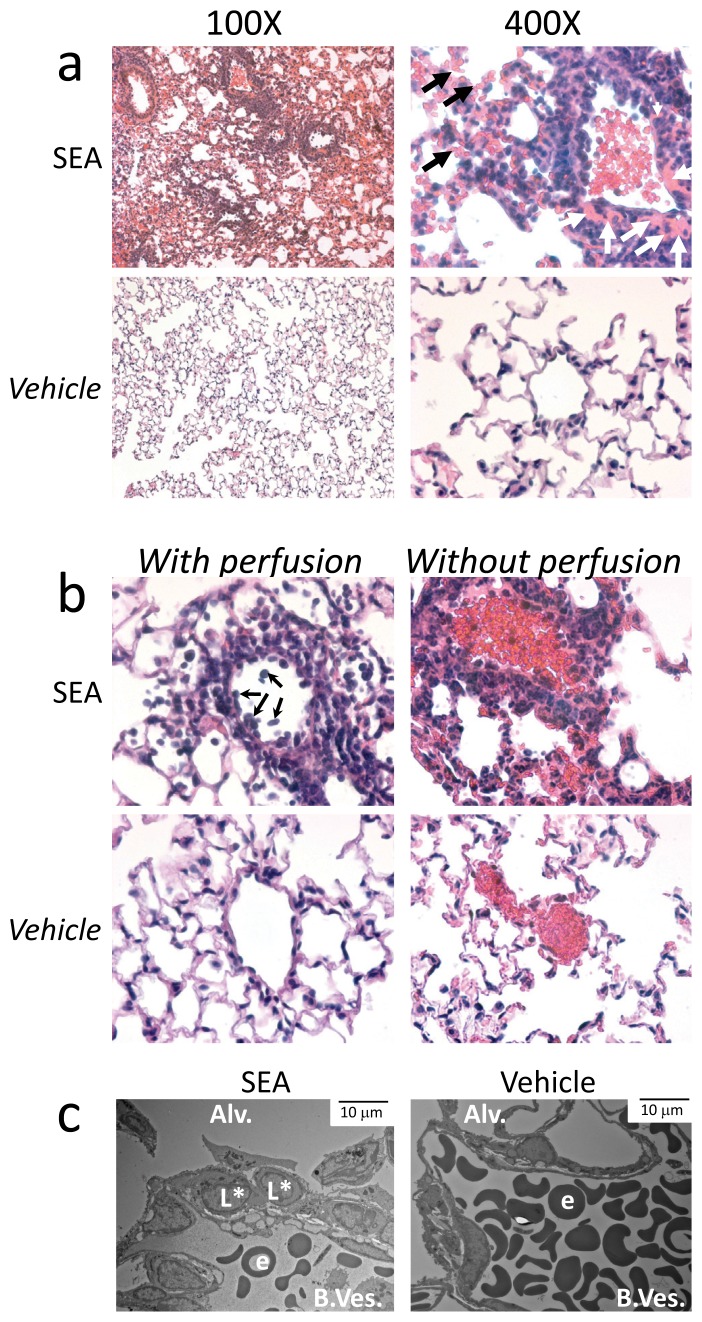
Intranasal SEA challenge induces lung injury and leukocyte infiltrate. Mice received 1 *µ*g of SEA diluted in BSS (Vehicle) through the i.n. route. (A) Lungs from day 2 SEA challenged mice were isolated, sectioned, and stained with H&E. Left panels display a low magnification (100X) and right panels high magnification (400X) of representative lung sections. Black arrows indicate leakage of red blood cells, white arrows indicate eosin staining refractile material (protein). (B) High magnification (400X) of lungs with (left panel) and without perfusion (right panel) of representative lung sections after vehicle alone and SEA i.n inhalation. Arrows indicate infiltrating leukocytes retained in blood vessel after perfusion. (C) Electron microscopy of lung blood vessels after vehicle alone and SEA i.n inhalation: Alveoli (Alv), Blood Vessel (B.Ves.), Erythrocyte (e), Leukocyte (L), Transmigrating leukocyte (L*). Bar  =  10 µm. The data are representative of three independent experiments using 2–4 mice per group.

We used an *in vivo* mouse model of ALI to uncover molecular biomarkers of lung inflammation. After inhalation of SEA, mice were monitored for signs of acute inflammation. In the current study we first examined lung pathology 2 days post SEA challenge, a time point where T cell effectors start to accumulate [11]. Lung sections stained by hematoxylin and eosin (H&E) showed leakage of red blood cells from blood vessels into the alveoli (Fig. 1A, black arrows in upper right panel), abundant leukocyte infiltration in the peri-vascular tissue, and increased cellularity in the interstitium and alveolar space as we observed previously [11]. Proteinous exudate was visible after SEA (Fig. 1A, white arrows in upper right panel). In contrast, lungs from vehicle-treated mice showed no signs of injury (Fig. 1A lower panels). To visualize the possibility of increased infiltrating leukocytes we performed the same inhalation study with or without lung perfusion. We found that perfusing the lung allowed for visualization of adherent leukocytes in the lumen of blood vessels after SEA treatment in the lung sections (Fig. 1B). In the absence of inflammation (vehicle) leukocyte adhesion was not evident. Secondly, electron microscopy (EM) was used to demonstrate the binding of leukocytes to the vascular lung endothelium. EM showed increased leukocyte adherence to blood vessel endothelium in the lung airways after SEA inhalation whereas vehicle alone injected mice showed no sign of leukocyte infiltration or adhesion to the endothelium (Fig. 1C). Moreover, leukocytes appeared to be transmigrating from the vasculature into lung tissue (Fig. 1C, left panel: L*). Altogether, these pathologic changes are consistent with rapid inflammation and immune-mediated lung damage.

**Figure 2:**
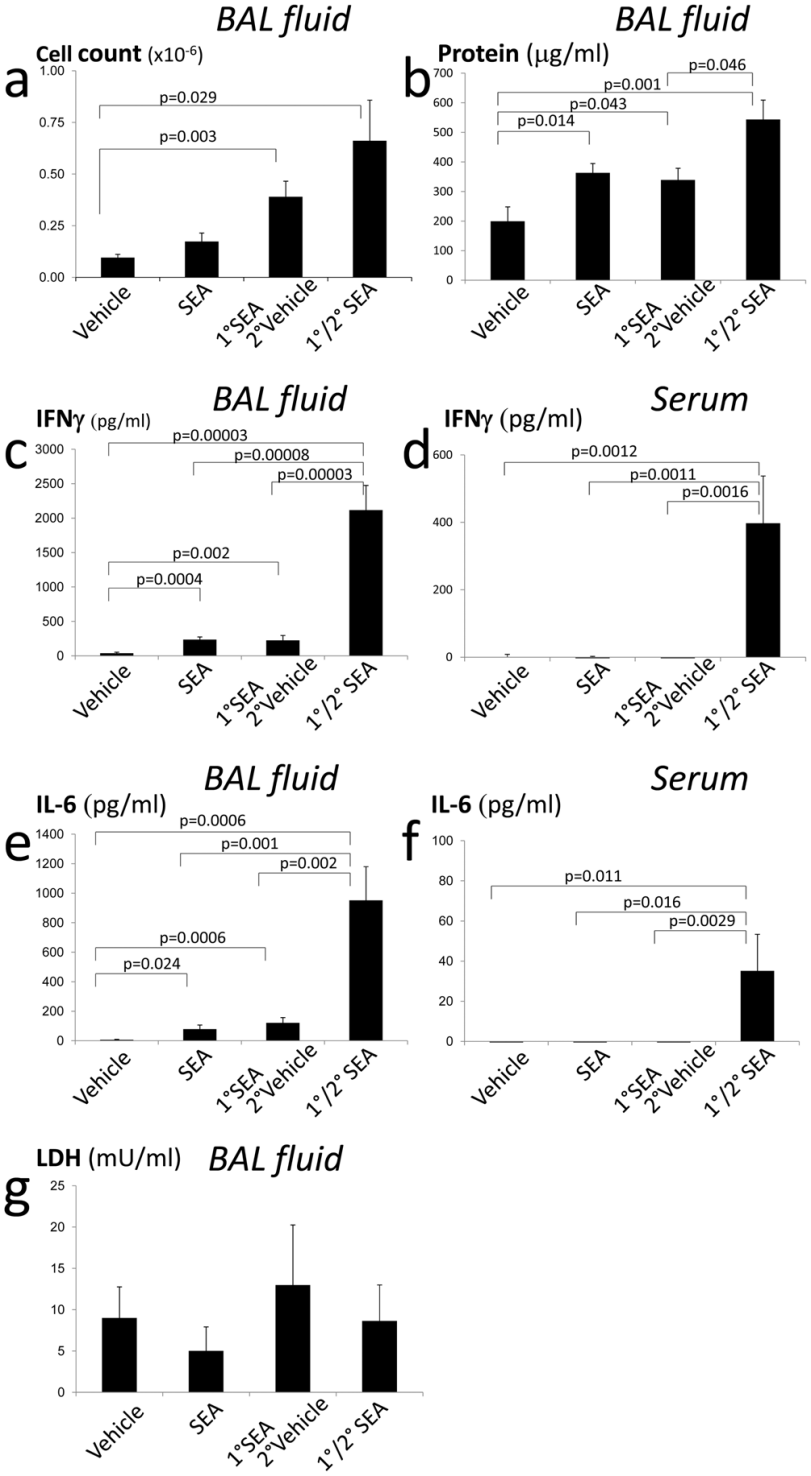
Intranasal SEA challenge induces cellular infiltration, protein leakage and inflammation. Mice received 1 *µ*g of SEA or vehicle alone i.n., secondary (2°) challenge of either vehicle or SEA 48 h after the primary (1°) and were sacrificed 5 h later (53 hrs total). (A) Cells present in BAL fluid were harvested and enumerated and (B) total BAL fluid protein quantified by BCA. (C) IFNγ in BAL fluid and (D) in serum, and (E) IL-6 in BAL and (F) in serum were quantified by ELISA. (G) LDH in BAL fluid was quantified. The data are representative of two independent experiments using 4 mice per group. The errors bars indicate the standard error of the mean between biological replicates.

Next, we measured parameters representative of ALI and tested the effect of 2° inhalation of SEA at inducing compounded inflammation. Increased cell number and total protein in BAL was evident (Figs. 2A and 2B). High levels of IFNγ and IL-6 were detected in BAL fluid after SEA inhalation and were strongly augmented after 2° SEA (Figs. 2C and E). Additionally, IFNγ and IL-6 were detected in serum demonstrating a powerful systemic response, which is a key factor in modeling ALI (Figs. 2D and F). Finally, lactate dehydrogenases (LDH), a marker of cell injury detectable in BAL fluid [16], [17] was present in BAL fluid but there was no difference after SEA inhalation (Fig. 2G). In fact, this result reinforces the current consensus for the need of new and more sensitive biochemical markers for ALI [1], [2].

### Identification of Early Markers of ALI

**Figure 3:**
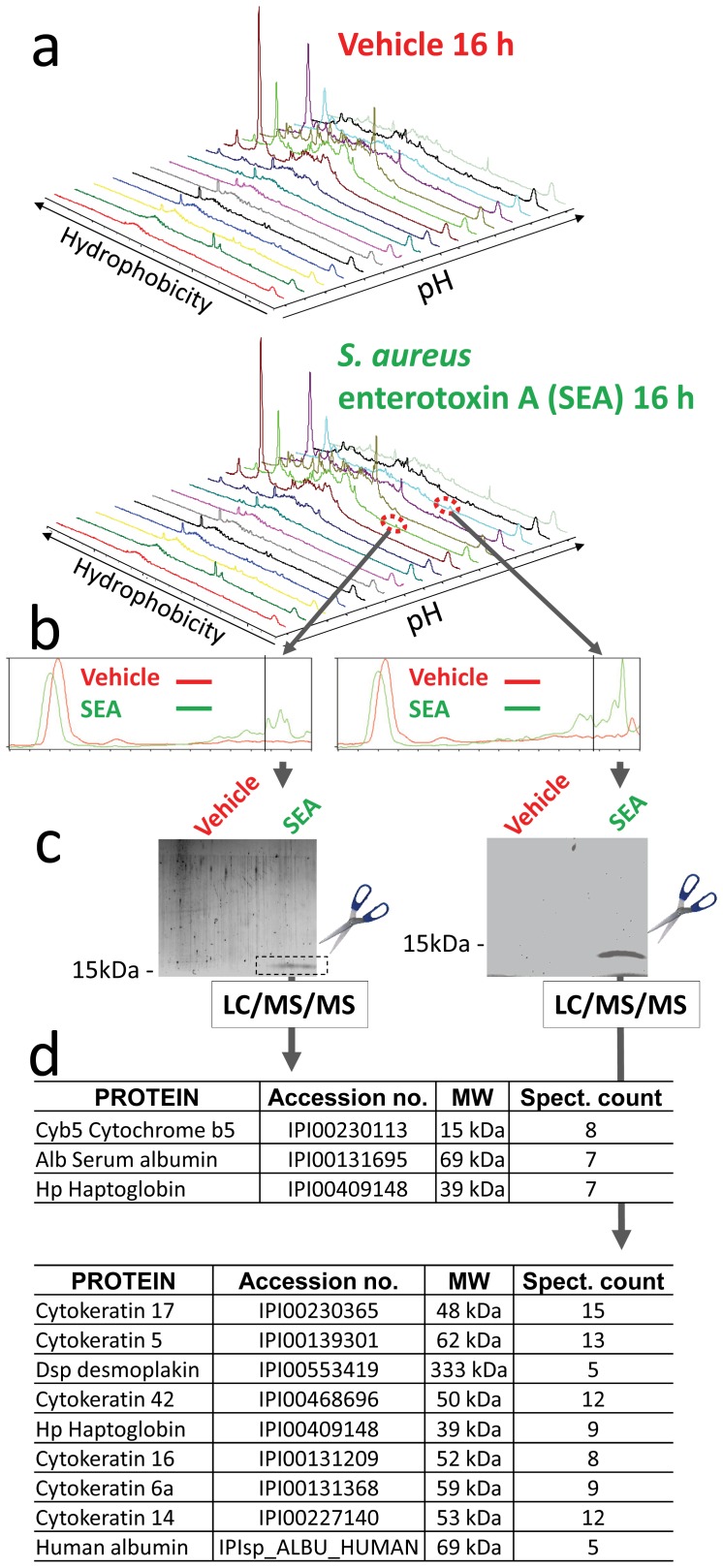
Differential proteomic fingerprint of BAL fluid from SEA *vs*. vehicle alone injected mice. Mice were immunized as described in the legend of figure 1. After 16 h BAL fluid was obtained and 2 mg of protein per sample was processed on the Beckman Coulter ProteomeLab PF 2D platform. Chromatofocusing was performed as a linear gradient from pH 8.0 to pH 4.0. Fractions were collected in 0.3 pH intervals, automatically reinjected for a second dimension on a C18 column at 50°C. (A) Two-dimensional proteomic maps of BAL fluid from SEA and vehicle alone immunized mice, representative of one out three experiments is shown. (B) Chromatograms of second dimension fractions from SEA treated mice were overlaid with their corresponding equivalents from vehicle alone-injected mice. Overlays revealed peaks present in two fractions of the SEA treated samples but undetectable in the vehicle alone injected samples. (C) The fractions were lyophilized and resolved by 4–15% SDS-PAGE. Bands detected by a protein-specific fluorescent dye were cut out, digested by trypsin, and identified by LC/MS/MS. (D) Peptides sequences were searched against the NCBInr database version 20060804 using the Proteometrics Software Suite and the Profound Search Algorithm. The data are representative of three independent biological replicates. Each sample run on the PF 2D was a pool of BAL fluid obtained from 5 mice.

To identify biomarkers of lung injury we used a proteomic approach comparing BAL fluid of SEA *versus* vehicle alone treated mice. Importantly, in order to find early biomarkers of ALI we collected BAL fluid 16 h after SEA inhalation and analyzed the samples using a PF 2D proteomics platform as described before [12], [15]. PF 2D proteomics enables protein separation by charge followed by reverse-phase chromatography (Fig. 3). Proteomic maps of BAL fluid SEA *versus* vehicle alone were similar as illustrated (Fig. 3A), but 2 peaks were spotted as differential fingerprints (Fig. 3A dotted circle). After careful analysis of three independent experiments using Proteoview software, two fingerprints were consistently identified at specific coordinates present in the BAL fluid map from 16 h SEA treated mice but absent or reduced in vehicle alone samples (Fig. 3B, right boxes). To identify the proteins contained in the differential fingerprint fractions, a third dimension was used. The fractions of interest were lyophilized, resolved by SDS-PAGE, and detected by fluorescent dye staining (Fig. 3C). The bands uniquely detected in the SEA BAL fluid samples were cut out of the gel, digested with trypsin and proteins identified by LC/MS/MS (Fig. 3D). Using this strategy cytochrome b5 and cytokeratin 17 were identified as differentially present upon SEA inhalation. Importantly, the sequences of cytochrome b5 and of cytokeratin 17 indicated the proteins were derived from mouse and were not contaminants of human origin. Interestingly, the molecular weight of cytochrome b5 corresponds to the migrating size (15 kDa) of the band on SDS-PAGE suggesting that the full length protein was isolated (Fig. 3D). All the other identified proteins had a higher molecular weight suggesting a breakdown product of these proteins were isolated. Several proteins like albumin and haptoglobin, known to be associated with inflammation and injury [18], [19] serve as a validation component of our analysis.

### Cytochrome b5 is a Marker of Lung Damage

**Figure 4:**
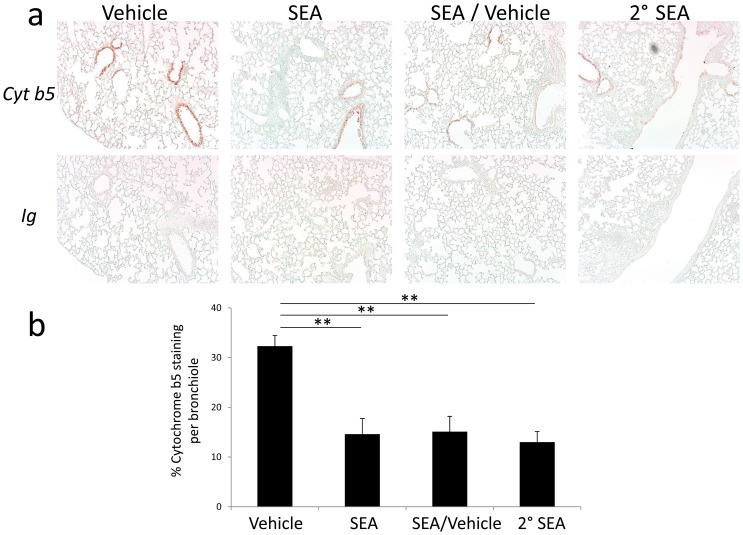
Cytochrome b5 expression is restricted to bronchiole epithelium in lungs. Mice were immunized i.n. with 1 *µ*g of SEA or vehicle alone i.n., secondary (2°) challenge of either vehicle or SEA 48 h after the primary, and were sacrificed 5 h later (53 hrs total). (A) Immunostaining of the lungs was performed using a rabbit polyclonal antibody against cytochrome b5 (Cyt b5) and rabbit immunoglobulin control (Ig). The data are representative of three independent experiments with 2–4 mice per group, magnification 100X. (B) The percentage of positive cytochrome b5 staining per bronchiole is reported for each sample by analyzing 30 representative bronchioles from two separate experiments. The errors bars indicate the standard error of the mean. **p<0.001.

We performed immunohistochemistry analysis of the proteins with the greatest spectral count as assessed by mass spectrometry (Fig. 3D) to validate their expression and to pinpoint from which site in the lung these proteins were derived. Staining for cytochrome b5 showed strong expression restricted to the epithelium of lung airways (Fig. 4A, top panels). Interestingly, the staining was quite evident in the airways of the vehicle alone treated group (Fig. 4A upper left panel), but weaker and punctuated in the 3 groups that received SEA (Fig. 4A the three upper right panels). This observation is consistent with the notion that cytochrome b5 may be released from epithelial cells of lung airways that are damaged locally by activated immune cells following SEA inhalation. To provide support for this notion, cytochrome b5 staining intensity was determined using Image-Pro Plus software and the data showed a significant decrease of staining in all groups that received SEA *versus* vehicle alone (Fig. 4B).

**Figure 5:**
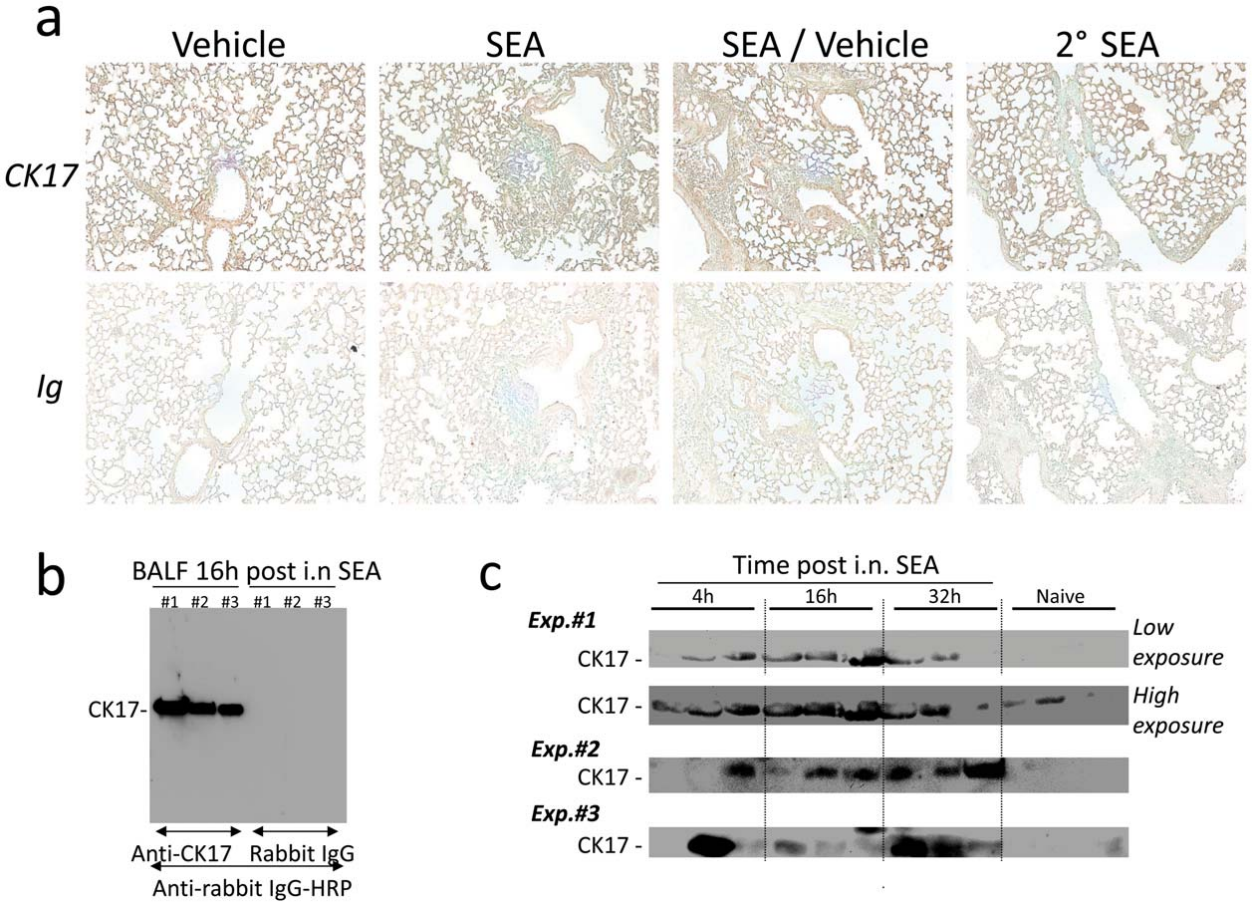
Detection of cytokeratin 17 in BAL fluid after i.n. SEA. Mice were immunized i.n. as described in legend of figure 4. (A) Immunostaining of the lungs was performed using a rabbit polyclonal antibody against cytokeratin 17 (CK17) and rabbit immunoglobulin control (Ig). The data are representative of three independent experiments, magnification 100X. (B) Immunoblotting of 5x concentrated BAL fluid was performed using a rabbit polyclonal against cytokeratin 17 (CK17) and rabbit IgG control. (C) BAL fluid harvested at different times after i.n. SEA immunization and BAL fluid from naïve mice were immunoblotted using anti- CK17 antibody. High exposure of the immunoblot revealed the presence of cytokeratin 17 in most samples including the samples harvested from naïve mice but with less intensity. The data show 3 separate experiments using with 3 mice per time point for each experiment.

We also validated the expression of cytokeratin 17 that is ubiquitously expressed in lung tissue, but contrary to cytochrome b5, the tissue expression of cytokeratin 17 in the lung was not visibly altered by SEA inhalation (Fig. 5a). We further validated the presence of cytokeratin 17 using immunoblot (Fig. 5b). Cytokeratin 17 accumulated in BAL fluid as early as 4 h post-SEA (Fig 5c) and a longer exposure of the membrane revealed cytokeratin 17 in BAL fluid from naïve mice (Fig 5c) and in vehicle alone BAL fluid (Fig. S1). Thus, cytokeratin 17 is sensitive to perturbations in lung. Immunoblotting for cytochrome b5 was less successful since a 60 kDa, instead of a 15 kDa, band was routinely detected which might have been a concatemer of cytochrome b5 as previously published (not shown) [20]. Altogether our data raise the possibility that cytochrome b5 and cytokeratin 17 are potential biomarkers of lung injury released from damaged epithelium early during pulmonary inflammation.

## Discussion

Using a proteomic-based approach we compared proteomic maps between BAL fluid from SEA *versus* vehicle-challenged mice and uncovered the presence of cytochrome b5 right after the onset of inflammation. Cytochrome b5 expression was restricted to epithelial cells of the bronchioles and SEA inhalation decreased the intensity of its signal. We propose that inflammation caused by cell injury mediates rapid release of cytochrome b5 into the extracellular space. Alternatively, cellular cytochrome b5 may be shed in the early stages of lung injury and found in the BAL fluid. Hence, cytochrome b5 is a potential biomarker of early lung injury.

Previous studies, and the model presented in this report, demonstrate that enterotoxin inhalation induces rapid T cell and innate cell activation that mimic the characteristics of human lung injury. These include acute onset of disease, obstruction of the airways [11], [21], [22], increased vascular permeability (Fig. 1) [22], endothelial and epithelial damage [21] (Fig. 1), production of nitric oxide [23], and increase protein and cytokine concentration in the lungs [11], [21], [22], [23] (Fig. 2). Hence, SEA inhalation in mouse provides a useful animal model of ALI that complements other experimental approaches [7]. In this report we specifically show rapid onset of protein deposition, leakage of red blood cells, increased adhesion of leukocytes in small blood vessels, leukocyte infiltration, and accumulation of proteins and cytokines in BAL fluid (Figs. 1–2). These data firmly establish that SEA inhalation can induce ALI.

The search for biomarkers of lung injury has provided candidates expressed in different anatomical compartments [24]. The von Willebrand factor (vWF) and ICAM-1 have been proposed as predictive markers of endothelial injury but with mixed results [25], [26], [27]. Surfactant proteins SP-A and SP-D expressed by type II pneumocytes, the receptor for advance glycation end-product (RAGE), and KL-6 have been associated with pulmonary epithelial cell damage [28], [29], [30], [31]. Desomosine, a stable fragment of elastine, has been used as a marker for destroyed extracellular matrix in ALI [32], [33]. Moreover, the presence of inflammatory cytokines present during ALI is often associated with a negative prognosis. For example, IL-1β, IL-6, IL-8, and IL-10 are associated with morbidity and mortality in ALI patients [34], [35]. However, the etiology of the disease and the presence of infectious agents promoting inflammation add to the complexity of finding the appropriate biomarker [25]. Although biomarkers of ALI have been identified, no single biomarker has been successfully used to diagnose and predict the clinical course of the disease [1], [2]. However, the combination of a panel of multiple biomarkers has been shown to be an improvement for predicting mortality in acute lung injury [36] and reinforces the idea that finding additional new early biomarkers will increase the accuracy of diagnosis.

To better understand the onset of lung injury the PF 2D was used to detect proteins in BAL fluid. Comparative proteomic mapping of BAL fluid taken from mice after SEA inhalation uncovered cytochrome b5 as a putative biomarker. Among the different isoforms of cytochrome b5, we identified the isoform bound to the ER membrane, called microsomal cytochrome b5 [37]. Following PF 2D fractionation, LC/MS/MS resolved 8 unique peptides covering 43% of microsomal cytochrome b5 sequence with perfect homology. However, a truncated soluble form of cytochrome b5 encoded by a second gene lacking the membrane binding carboxy terminus and expressed in erythrocytes, lung, gallbladder, adrenal gland, and bone marrow [38] matches the peptide sequences. Nevertheless, the molecular size of the protein isolated by PF 2D fractionation corresponded to the membrane bound microsomal and not soluble cytochrome b5. Additional genetic studies will be needed to confirm with absolute certainty which form of cytochrome b5 has been identified in our study. However, we ruled out OMb, the mitochrondrial isoform of cytochrome b5 [39], which has only 58% amino acid conservation with microsomal cytochrome b5 [40]. Cytochrome b5 is a highly conserved protein involved in electron transfer between NADH/NADPH and cytochrome P450, participating in the oxidation of a wide array of endogenous and xenobiotic substances [37], [41]. The use of model substrates and drugs in conditional hepatic knock-out cytochrome b5 mice demonstrated that P450-mediated metabolism is dependent on cytochrome b5 [42]. Interestingly, systemic LPS administration decreases cytochrome P450 mRNA expression in lungs [43], which support the finding that inhibitors of cytochrome P450 activity exacerbate LPS-mediated inflammation while compounds known to induce cytochrome P450 reduce inflammation [44]. Thus, cytochrome b5 may influence inflammatory responses by impacting P450 function. Additionally, some of the immunomodulatory proteins released from lung epithelial cells, such as CC16 [45], surfactant proteins [46], [47], and galectin-3 [48] are considered damage-associated molecular-patterns (DAMPs) [49]. Therefore, cellular damage could induce release of DAMPs and aggravate ALI. We identified cytochrome b5 and cytokeratin 17 by focusing on the most prominent and reproducible differences apparent on our proteomic maps. However, it is possible that known DAMPs were present in the BAL fluid but not identified.

Some of the biomarkers identified in our study could potentially be DAMPs and worsen lung injury by promoting the inflammatory response. For example, cytokeratin 17, which we detected in the BAL fluid after SEA inhalation, can trigger cytokine production and inflammation *in vivo* [50]. Thus, the presence of proteins in BAL fluid after SEA inhalation may enhance lung inflammation. To the best of our knowledge the immunomodulatory effect of cytochrome b5 is unknown, but our results show that cytochrome b5 is modulated during the early stages of lung cell injury and perhaps represent a new target for therapeutic intervention.

## Materials and Methods

### Mice

C57BL/6 mice were purchased from the National Cancer Institute (Frederick, MD) or the Jackson Laboratory (Bar Harbor, ME). All mice were maintained in the central animal facility at the University of Connecticut Health Center (UCHC) in accordance with federal guidelines. The present study was approved by the University of Connecticut Health Center’s Animal Care Committee.

### Antibodies and ELISA

IFNγ and IL-6 ELISA kits were purchased from Pharmingen (Mountain View, CA). Anti-cytochrome b5 rabbit polyclonal antibody was purchased from Abcam (Cambridge, MA). Anti-cytokeratin 17 rabbit polyclonal antibody was purchased from Santa Cruz Biotechnology (Santa Cruz, CA). LDH detection kit was purchased from Cell Sciences (Canton, MA).

### Microscopy

PBS/heparin solution was used for perfusion. Lungs were inflated with 10% formalin solution, clamped for 5 min, fixed for 2 h and stored in 70% ethanol before paraffin embedding. Lung sections were stained for H&E or stained with the appropriate antibody for immunohistochemistry (IHC) and counter stained with methyl green (Vector laboratory, Inc. Burlingame, CA). The percentage of bronchiole cells staining positive for cytochrome b5 was quantified using Image-Pro Plus software (Media Cybernetics, Inc. Bethesda, MD). Pixel analysis was performed on 30 representative bronchioles for each condition. Data was reported as the percentage of positive staining area (brown) *versus* total staining area (all colors) present in each bronchiole. Lung sections for Electron Microscopy were processed at the central microscope facility, University of Connecticut Health Center. Lungs were fixed in 2.5% glutaraldehyde in 0.1M cacodylate buffer pH 7.2, post fixed in 1% osmium tetroxide, 0.8% potassium ferricyanide in 0.1M cacodyalte buffer, en-bloc stained with uranyl acetate, dehydrated in ethanol and embedded in Polybed 812 resin. Thin sections (70 nm) of the lungs were cut and counterstained with uranyl acetate and lead citrate. Thin sections were examined using a Hitachi H-7650 transmission electron microscope.

### BAL Fluid Processing and PF 2D Proteomics

BAL was collected by lavage of mouse lung with 5 ml of sterile PBS. BAL was centrifuged at 1,000 rpm at 4°C to separate cells from BAL fluid. BAL fluid was spiked with a protease inhibitor cocktail (SIGMA #P2714), centrifuged at 25,000 x g, 4°C for 10 min, protein concentration was determined by BCA assay (Pierce, Rockford, IL), and processed through a Beckman Coulter ProteomeLab™ PF 2D platform (Fullerton, CA) as described before [12], [15], [51]. Fractions were collected every 0.3 pH units. Fractions corresponding to the linear gradient between pH 8.0 through 4.0 were separated on a HPRP column (Beckman Coulter) with a gradient from 0 to 100% of acetonitrile at 50°C. The proteins were detected with UV light at 214 nm and collected at 0.5 min intervals and stored at −80°C.

### Two Dimensional (2D) Protein Map Analysis

Two dimensional protein expression maps of BAL fluid displaying protein isoelectric point (pI) *versus* protein hydrophobicity, were generated by the ProteoView/DeltaVue software package as described earlier [12], [15].

### SDS-PAGE, Immunoblotting, Fluorescence Staining, and LC/MS/MS

The PF 2D fractions of interest were lyophilized and resuspended in denaturing SDS sample buffer and resolved by SDS-PAGE and immunoblotting as described earlier [15]. Gels were stained by ORIOLE (BioRad, Hercules, CA) and proteins were detected by fluorescence. Visible bands were excised from the gel, subjected to tryspin digestion and identified by LC/MS/MS at NextGen Sciences (Ann Arbor, MI). Tandem mass spectra were extracted by Xcalibur (ThermoFisher) rev. 2.0. All MS/MS samples were analyzed using Mascot version 1.0 (Matrix Science, London, UK; version Mascot) assuming digestion by the enzyme trypsin. Protein identifications were accepted if they could be established at greater than 90.0% probability by the Protein Prophet algorithm [52] and contained at least two identified peptides.

## Supporting Information

Figure S1 **Detection of cytokeratin 17 in BAL fluid after i.n. SEA and BSS.** Mice were immunized i.n. as described in legend of figure 4 and BAL fluid harvested after 16 h. BAL fluid were immunoblotted using anti-CK17 antibody. Data are from 1 experiment.
(TIF)

## References

[1] Kuebler WM (2011). Biomarkers of acute respiratory distress syndrome: Do good things lie nearby?. Crit Care Med.

[2] Gaggar A, Olman MA (2006). Biologic markers of mortality in acute lung injury.. Clin Chim Acta.

[3] Matute-Bello G, Downey G, Moore BB, Groshong SD, Matthay MA (2011). An official American Thoracic Society workshop report: features and measurements of experimental acute lung injury in animals.. Am J Respir Cell Mol Biol.

[4] Thomas D, Chou S, Dauwalder O, Lina G (2007). Diversity in Staphylococcus aureus enterotoxins.. Chem Immunol Allergy.

[5] Iscimen R, Cartin-Ceba R, Yilmaz M, Khan H, Hubmayr RD (2008). Risk factors for the development of acute lung injury in patients with septic shock: an observational cohort study.. Crit Care Med.

[6] Johnson ER, Matthay MA (2010). Acute lung injury: epidemiology, pathogenesis, and treatment.. J Aerosol Med Pulm Drug Deliv.

[7] Matute-Bello G, Frevert CW, Martin TR (2008). Animal models of acute lung injury.. Am J Physiol Lung Cell Mol Physiol.

[8] Bachert C, Gevaert P, Zhang N, van Zele T, Perez-Novo C (2007). Role of staphylococcal superantigens in airway disease.. Chem Immunol Allergy.

[9] Tripathi A, Kern R, Conley DB, Seiberling K, Klemens JC (2005). Staphylococcal exotoxins and nasal polyposis: analysis of systemic and local responses.. Am J Rhinol.

[10] Kim ST, Chung SW, Jung JH, Ha JS, Kang IG (2011). Association of T cells and eosinophils with Staphylococcus aureus exotoxin A and toxic shock syndrome toxin 1 in nasal polyps.. Am J Rhinol Allergy.

[11] Muralimohan G, Rossi RJ, Guernsey LA, Thrall RS, Vella AT (2008). Inhalation of Staphylococcus aureus enterotoxin A induces IFN-gamma and CD8 T cell-dependent airway and interstitial lung pathology in mice.. J Immunol.

[12] Nakanishi M, Menoret A, Belinsky GS, Giardina C, Godman CA (2007). Utilizing endoscopic technology to reveal real-time proteomic alterations in response to chemoprevention.. Proteomics Clin Appl.

[13] Beckhove P, Warta R, Lemke B, Stoycheva D, Momburg F (2010). Rapid T cell-based identification of human tumor tissue antigens by automated two-dimensional protein fractionation.. J Clin Invest.

[14] Lee HJ, Kang MJ, Lee EY, Cho SY, Kim H (2008). Application of a peptide-based PF2D platform for quantitative proteomics in disease biomarker discovery.. Proteomics.

[15] Menoret A, McAleer JP, Ngoi SM, Ray S, Eddy NA (2009). The oxazolidinone derivative locostatin induces cytokine appeasement.. J Immunol.

[16] Han SG, Andrews R, Gairola CG, Bhalla DK (2008). Acute pulmonary effects of combined exposure to carbon nanotubes and ozone in mice.. Inhal Toxicol.

[17] Mantecca P, Farina F, Moschini E, Gallinotti D, Gualtieri M (2010). Comparative acute lung inflammation induced by atmospheric PM and size-fractionated tire particles.. Toxicol Lett.

[18] Dobryszycka W (1997). Biological functions of haptoglobin–new pieces to an old puzzle.. Eur J Clin Chem Clin Biochem.

[19] Engstrom G, Segelstorm N, Ekberg-Aronsson M, Nilsson PM, Lindgarde F (2009). Plasma markers of inflammation and incidence of hospitalisations for COPD: results from a population-based cohort study.. Thorax.

[20] Lombard N, Swart AC, van der Merwe MJ, Swart P (2002). Sheep adrenal cytochrome b5: active as a monomer or a tetramer in vivo?. Endocr Res.

[21] LeClaire RD, Hunt RE, Bavari S, Estep JE, Nelson GO (1996). Potentiation of inhaled staphylococcal enterotoxin B-induced toxicity by lipopolysaccharide in mice.. Toxicol Pathol.

[22] Rieder SA, Nagarkatti P, Nagarkatti M (2011). CD1d-Independent Activation of Invariant Natural Killer T Cells by Staphylococcal Enterotoxin B through Major Histocompatibility Complex Class II/T Cell Receptor Interaction Results in Acute Lung Injury.. Infect Immun.

[23] Miyakawa H, Sato K, Shinbori T, Okamoto T, Gushima Y (2002). Effects of inducible nitric oxide synthase and xanthine oxidase inhibitors on SEB-induced interstitial pneumonia in mice.. Eur Respir J.

[24] Levitt JE, Gould MK, Ware LB, Matthay MA (2009). The pathogenetic and prognostic value of biologic markers in acute lung injury.. J Intensive Care Med.

[25] Calfee CS, Eisner MD, Ware LB, Thompson BT, Parsons PE (2007). Trauma-associated lung injury differs clinically and biologically from acute lung injury due to other clinical disorders.. Crit Care Med.

[26] Bajaj MS, Tricomi SM (1999). Plasma levels of the three endothelial-specific proteins von Willebrand factor, tissue factor pathway inhibitor, and thrombomodulin do not predict the development of acute respiratory distress syndrome.. Intensive Care Med.

[27] Ware LB, Eisner MD, Thompson BT, Parsons PE, Matthay MA (2004). Significance of von Willebrand factor in septic and nonseptic patients with acute lung injury.. Am J Respir Crit Care Med.

[28] Ishizaka A, Matsuda T, Albertine KH, Koh H, Tasaka S (2004). Elevation of KL-6, a lung epithelial cell marker, in plasma and epithelial lining fluid in acute respiratory distress syndrome.. Am J Physiol Lung Cell Mol Physiol.

[29] Calfee CS, Ware LB, Eisner MD, Parsons PE, Thompson BT (2008). Plasma receptor for advanced glycation end products and clinical outcomes in acute lung injury.. Thorax.

[30] Cheng IW, Ware LB, Greene KE, Nuckton TJ, Eisner MD (2003). Prognostic value of surfactant proteins A and D in patients with acute lung injury.. Crit Care Med.

[31] Eisner MD, Parsons P, Matthay MA, Ware L, Greene K (2003). Plasma surfactant protein levels and clinical outcomes in patients with acute lung injury.. Thorax.

[32] Kropski JA, Fremont RD, Calfee CS, Ware LB (2009). Clara cell protein (CC16), a marker of lung epithelial injury, is decreased in plasma and pulmonary edema fluid from patients with acute lung injury.. Chest.

[33] Tenholder MF, Rajagopal KR, Phillips YY, Dillard TA, Bennett LL (1991). Urinary desmosine excretion as a marker of lung injury in the adult respiratory distress syndrome.. Chest.

[34] Parsons PE, Eisner MD, Thompson BT, Matthay MA, Ancukiewicz M (2005). Lower tidal volume ventilation and plasma cytokine markers of inflammation in patients with acute lung injury.. Crit Care Med 33: 1–6; discussion 230–232.

[35] Meduri GU, Headley S, Kohler G, Stentz F, Tolley E (1995). Persistent elevation of inflammatory cytokines predicts a poor outcome in ARDS. Plasma IL-1 beta and IL-6 levels are consistent and efficient predictors of outcome over time.. Chest.

[36] Calfee CS, Ware LB, Glidden DV, Eisner MD, Parsons PE (2011). Use of risk reclassification with multiple biomarkers improves mortality prediction in acute lung injury.. Crit Care Med.

[37] Schenkman JB, Jansson I (2003). The many roles of cytochrome b5.. Pharmacol Ther.

[38] Giordano SJ, Steggles AW (1993). Differential expression of the mRNAs for the soluble and membrane-bound forms of rabbit cytochrome b5.. Biochim Biophys Acta.

[39] Lederer F, Ghrir R, Guiard B, Cortial S, Ito A (1983). Two homologous cytochromes b5 in a single cell.. Eur J Biochem.

[40] Vergeres G, Waskell L (1995). Cytochrome b5, its functions, structure and membrane topology.. Biochimie.

[41] Durr UH, Waskell L, Ramamoorthy A (2007). The cytochromes P450 and b5 and their reductases–promising targets for structural studies by advanced solid-state NMR spectroscopy.. Biochim Biophys Acta.

[42] Finn RD, McLaughlin LA, Ronseaux S, Rosewell I, Houston JB (2008). Defining the in Vivo Role for cytochrome b5 in cytochrome P450 function through the conditional hepatic deletion of microsomal cytochrome b5.. J Biol Chem.

[43] Theken KN, Deng Y, Kannon MA, Miller TM, Poloyac SM (2011). Activation of the acute inflammatory response alters cytochrome P450 expression and eicosanoid metabolism.. Drug Metab Dispos.

[44] Tesfaigzi Y, Kluger M, Kozak W (2001). Clinical and cellular effects of cytochrome P-450 modulators.. Respir Physiol.

[45] Hung CH, Chen LC, Zhang Z, Chowdhury B, Lee WL (2004). Regulation of TH2 responses by the pulmonary Clara cell secretory 10-kd protein.. J Allergy Clin Immunol.

[46] Chroneos ZC, Sever-Chroneos Z, Shepherd VL (2010). Pulmonary surfactant: an immunological perspective.. Cell Physiol Biochem.

[47] Madan T, Reid KB, Clark H, Singh M, Nayak A (2010). Susceptibility of mice genetically deficient in SP-A or SP-D gene to invasive pulmonary aspergillosis.. Mol Immunol.

[48] Henderson NC, Sethi T (2009). The regulation of inflammation by galectin-3.. Immunol Rev.

[49] Sato S, St-Pierre C, Bhaumik P, Nieminen J (2009). Galectins in innate immunity: dual functions of host soluble beta-galactoside-binding lectins as damage-associated molecular patterns (DAMPs) and as receptors for pathogen-associated molecular patterns (PAMPs).. Immunol Rev.

[50] Depianto D, Kerns ML, Dlugosz AA, Coulombe PA (2010). Keratin 17 promotes epithelial proliferation and tumor growth by polarizing the immune response in skin.. Nat Genet.

[51] Barre O, Solioz M (2006). Improved protocol for chromatofocusing on the ProteomeLab PF2D.. Proteomics.

[52] Keller A, Nesvizhskii AI, Kolker E, Aebersold R (2002). Empirical statistical model to estimate the accuracy of peptide identifications made by MS/MS and database search.. Anal Chem.

