# Supplementary figures and images for: Cytochrome b5 and Cytokeratin 17 Are Biomarkers in Bronchoalveolar Fluid Signifying Onset of Acute Lung Injury
Source: PLoS One. 2012 Jul 6;7(7):e40184. doi: 10.1371/journal.pone.0040184 (PMC3391234; doi:10.1371/journal.pone.0040184)

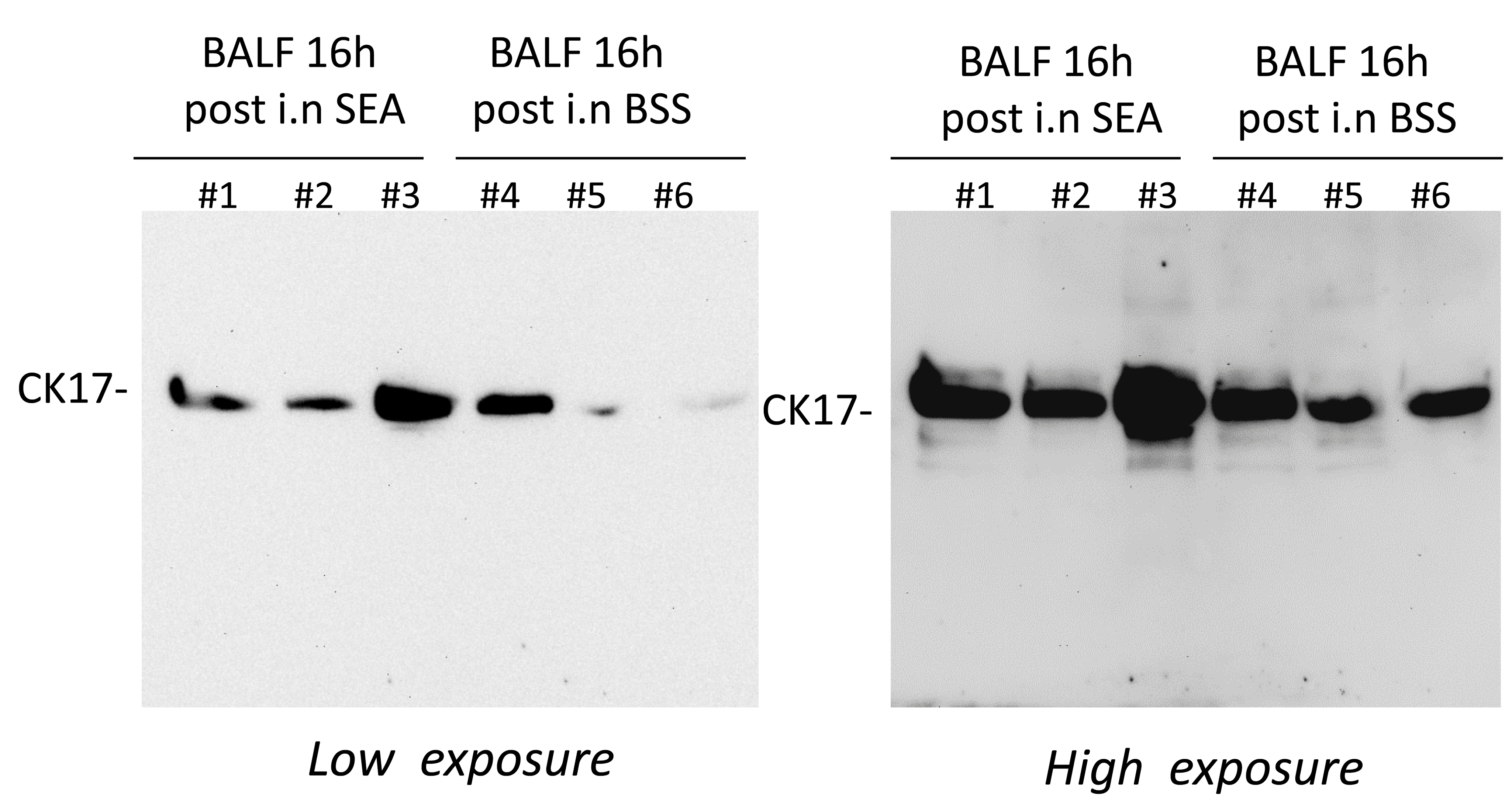

Supplement: Figure S1 — Detection of cytokeratin 17 in BAL fluid after i.n. SEA and BSS. Mice were immunized i.n. as described in legend of figure 4 and BAL fluid harvested after 16 h. BAL fluid were immunoblotted using anti-CK17 antibody. Data are from 1 experiment. (TIF) [file pone.0040184.s001.tif]
